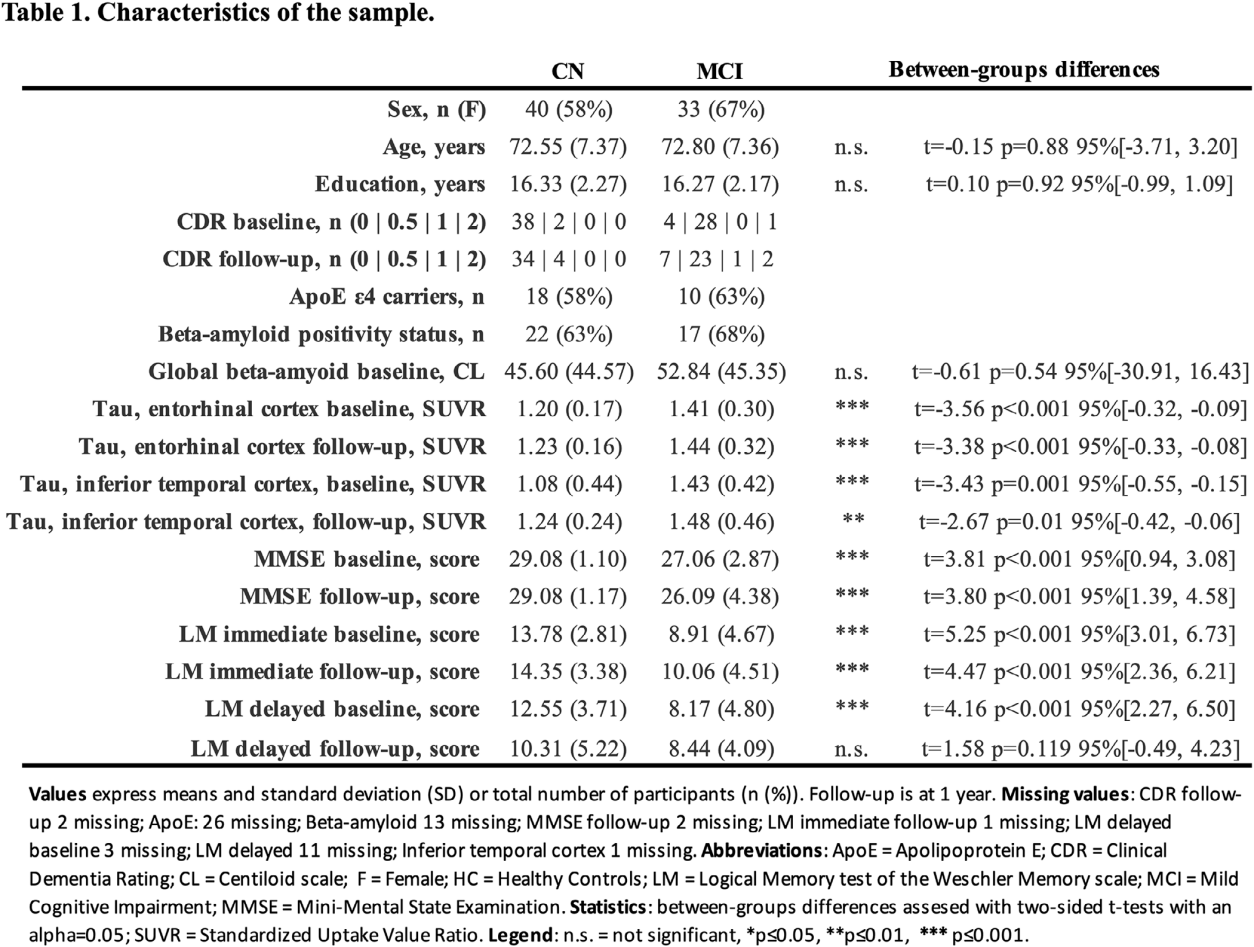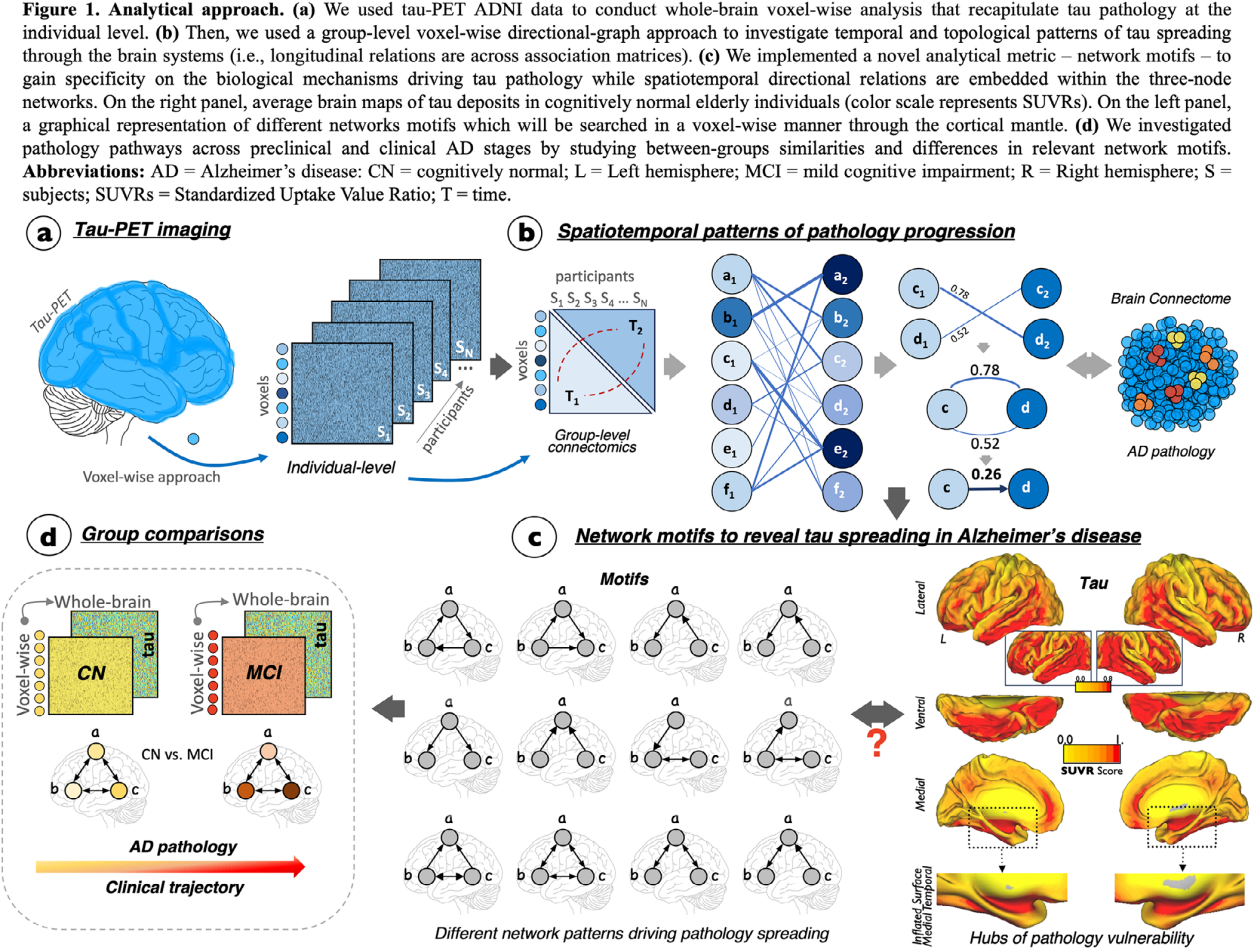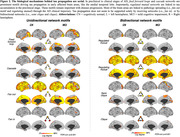# AD Pathology Spreading Detection Using Directional Motifs in Brain Connectomes

**DOI:** 10.1002/alz.088834

**Published:** 2025-01-03

**Authors:** Elisenda Bueichekú, Ibai Diez, Antonio Úbeda, Chan‐Mi Kim, Heidi I.L. Jacobs, Jorge Sepulcre

**Affiliations:** ^1^ Gordon Center for Medical Imaging, Massachusetts General Hospital, Boston, MA USA; ^2^ Harvard Medical School, Boston, MA USA; ^3^ Yale PET Center, New Haven, CT USA; ^4^ Yale University, New Haven, CT USA; ^5^ Massachusetts General Hospital, Boston, MA USA; ^6^ Athinoula A. Martinos Center for Biomedical Imaging, Charlestown, MA USA; ^7^ School of Medicine, Navarra Spain; ^8^ Department of Radiology, Massachusetts General Hospital, Harvard Medical School, Boston, MA USA; ^9^ Massachusetts General Hospital, Harvard Medical School, Boston, MA USA; ^10^ Athinoula A. Martinos Center for Biomedical Imaging, Department of Radiology, Massachusetts General Hospital, Harvard Medical School, Charlestown, MA USA; ^11^ Faculty of Health, Medicine and Life Sciences, Maastricht University, Maastricht Netherlands; ^12^ Yale PET Center, Yale Medical School, New Haven, CT USA

## Abstract

**Background:**

The accumulation of misfolded tau proteins, an Alzheimer’s disease (AD) hallmark, starts decades before the emergence of cognitive decline and clinical diagnosis. Autopsy studies support a predictable progression of tau pathology through large‐scale systems. However, less is known about the specific progression patterns. The use of connectomic metrics could enhance our understanding of the characteristics of pathology spread. We examined the spatiotemporal relationships of cortical tau accumulation using network motifs, directed graph metrics, which can reveal the underlying mechanisms governing the pathology progression routes in preclinical and clinical AD.

**Methods:**

We used longitudinal tau‐PET data (1‐year follow‐up) from 40 adults with normal cognitive functioning and 33 adults with mild cognitive impairment from the Alzheimer’s Disease Neuroimaging Initiative (ADNI‐3) (Table 1). We used individual and group‐level whole‐brain voxel‐wise graph theory and motifs networks analyses to investigate AD‐related tau pathology directional spreading (Figure 1a). Directional relations are established by using group‐level association matrices and by assessing differences in between‐voxels temporal relations (Time1‐Time2; FDR‐corrected, p<0.001) (Figure 1b). Then, the resulting association matrix representing the human connectome is decomposed into motif networks, which are three‐node triangular fundamental relations (i.e., exact copies of every possible motif is searched at the voxel‐level) (Figure 1c). Finally, between‐group visual comparisons of each motif reveal similarities and differences in dominant pathology spreading patterns in preclinical vs. clinical AD stages (Figure 1d).

**Results:**

Our results support that unidirectional and serial network motifs are behind misfolded tau protein spreading in AD (Figure 2). *Feed‐forward loops*, *cascade*, and *regulated mutual networks* drive tau propagation in medial and lateral temporal and medial prefrontal regions early in the disease. Through the disease, spread out from one region to another (i.e., *fan out* and *regulating mutual networks*) is a common characteristic of tau progression.

**Conclusion:**

Our results suggest that the progression of toxic tau through the brain systems occurs mainly in a unidirectional and serial manner. AD is considered a dysconnectivity disease. Thus, serial motifs could indicate biological relations arising on a sequential spatiotemporal scale. Future developments will examine the role of amyloid accumulation in tau progression in the context of AD.